# DATFAP: A Database of Primers and Homology Alignments for Transcription Factors from 13 Plant Species

**DOI:** 10.1186/1471-2164-9-140

**Published:** 2008-03-25

**Authors:** Jakob Fredslund

**Affiliations:** 1 BiRC – Bioinformatics Research Center, University of Aarhus, Høegh-Guldbergs Gade 10, Building 1090, DK-8000 Århus C, Denmark

## Abstract

**Background:**

Many well-known transcription factor databases do not provide PCR primers for the sequences. Often, transcription factors from the same family have a very high sequence similarity, and so specific primer sets that only amplify their targets and none of the other family members may be hard to design manually. Also, it may often be useful to have one general primer set targeting the slightly different homologs of some transcription factor from several species.

**Results:**

DATFAP is a free, web-based, very user-friendly browsing tool based on a new database of more than 55,000 EST sequences from 13 plant species, classified as transcription factors. Further, the database offers primers designed for RealTime-PCR as well as homology alignments and phylogenies for the sequences. The provided PCR primers are designed so that they have a perfect sequence alignment to their target only and not to any other sequences in the database from the same species. Via a direct link to a helper tool, the user may also design a general primer set targeting all sequences in any hand-picked group of homologs. A sophisticated search facility enables the user to find exactly the relevant sequences which subsequently may be easily downloaded. All homologies among the more than one billion possible pairwise sequence comparisons of DATFAP have been charted in advance. Thus, the user may quickly display the alignment of any sequence and all its database homologs.

**Conclusion:**

Because of the comprehensive homology analysis, it is very easy to find related transcription factors from different species, i.e. to navigate the network of inter-related transcription factors from the different species, and to find specific or general primers for them. DATFAP is found at the project homepage.

## Background

Transcription factors are proteins acting to regulate expression of genes by binding to their regulatory regions. Activated in signal transduction pathways, they are essential in the production of the right cellular response to some stimulus. Hence, specific transcription factors are the topic of study in hundreds of research projects, including projects dealing with plant breeding. Such studies can be very different in nature, ranging from the precise, functional characterization of certain proteins [1] over identification and classification of families of related proteins [2] to genetic modification [3].

When examining cDNA samples for the presence or expression levels of transcripts, one very often uses the Polymerase Chain Reaction, PCR. As many transcription factor families exist with minor variations among members, detecting one particular member requires uniquely specific PCR primers capable of binding to the target only, and avoiding the (often highly similar) relatives. Under other circumstances it might be relevant to have *one general *set of primers targeting a certain transcription factor such that the same primer set would amplify the correct target in several different species; a sort of orthogonal primer design problem.

In the search of new or unannotated transcription factors, sequence similarity to known transcription factors is a strong indicator. A large number of families have been identified, among plants in particular in rice and the model plant *Arabidopsis thaliana*, and homologs in other species to proteins from these families have been found.

This paper presents DATFAP (Database of Transcription Factors with Alignments and Primers), a resource where transcription factors from 12 plants and the green algae *Chlamydomonas reinhardtii *are collected and made available together with specific primers, homology alignments and sequence phylogenies.

## Construction and Content

The EST sequences in DATFAP originate primarily from the Institute of Genomic Research (consensus sequences of EST alignments [4]), but also from the National Center for Biotechnology Information [5], the Database of Arabidopsis Transcription Factors [6], PlnTFDB [7], and PlantTFDB [8]. A sequence is classified as a transcription factor in DATFAP if one or more of the following conditions are met:

• [keyword] The phrase "transcription factor" or one of the known transcription factor family names defined for rice or *Arabidopsis *is found in its header.

• [external database] It is listed in one of the above-mentioned external databases of transcription factors.

• [gene ontology] It has a Gene Ontology [9] annotation containing the phrase "transcription factor" or one of the known transcription factor family names defined for rice or *Arabidopsis*.

• [homology] It is very similar to a DATFAP sequence from another species classified by the above criteria as a transcription factor. Similarity is determined using a standalone version of the program BLAST [10] with *e*-value 10^-7^.

Besides its header, each sequence is associated with a **classification record **which states the reason(s) for its classification as a transcription factor in DATFAP.

The transcription factor family keywords used are: abi3, abi3/vp1, abi3vp1, alfin, alfin-like, ap2, ap2-erebp, ap2/erebp, arf, arid, arr-b, as2, at-hook, atrkd, aux, aux-iaa, bbr, bbr-bpc, bbr/bpc, bes1, bhlh, bpc, bzip, bzr, c2c2-co, c2c2-co-like, c2c2-dof, c2c2-gata, c2c2-yabby, c2h2, c3h, c3h-type 1, c3h-type 2, camta, cbf5, ccaat-dr1, ccaat-hap2, ccaat-hap3, ccaat-hap5, cpp, dbp, dp, e2f, e2f-dp, e2f/dp, eil, erebp, erf, fha, g2, g2-like, garp-arr-b, garp-g2-like, gata, gebp, gif, gras, grf, hb, hmg, hmg-box, homeobox, hrt, hrt-like, hsf, iaa, jumonji, lfy, lim, lim-domain, lug, mads, mbf1, myb, myb-related, nac, nin-like, nlp, nozzle, nsp1, nsp2, nzz, orphan, orphans, pbf-2-like, pcg, phd, phd-finger, platz, pseudo arr-b, pti4, pti5, pti6, rav, rem, rwp-rk, s1fa-like, sap, sbp, sigma70-like, sir2, srs, sw13, swi4/swi6, taz, tcp, tga3, tlp, trihelix, tub, ult, vip3, voz, voz-9, vp1, whirly, wrky, zf-hd, zim [6-8,11,12].

## Utility and Discussion

Following the above-mentioned criteria, 55,361 out of more than 750,000 downloaded sequences were classified as transcription factors and curated in DATFAP. The access point for DATFAP is the front page where the user gives directives to the search engine via input to four text boxes. First of all, in the leftmost selection box, one or more species must be chosen among

• *Medicago truncatula*

• *Zea mays *(maize)

• *Oryza sativa *(rice)

• *Lycopersicon esculentum *(tomato)

• *Chlamydomonas reinhardtii*

• *Solanum tuberosum *(potato)

• *Populus trichocarpa *(poplar)

• *Glycine max *(soybean)

• *Hordeum vulgare *(barley)

• *Lotus japonicus*

• *Arabidopsis thaliana*

• *Gossypium hirsutum *(cotton)

• *Triticum aestivum *(wheat)

In the text box entitled "Search keywords", the user may type any keyword(s) of particular interest, e.g. *homeobox*. It is also possible simply to type an ID such as *aw694210*. Only sequences whose headers or classification records contain at least one of the search keywords will be selected. The search is case-insensitive.

Complex search queries can be composed using OR, AND, NOT, *, single quotes and parentheses; e.g.:

'zinc finger' and ('purine-binding' or CCCH) and not (partial or putative)

Some further details about the search facility:

• Parentheses are used to build sub-expressions

• Single quotes are used around search words or phrases containing spaces

• The wildcard character * is used to search for *parts of *words. E.g., trans* matches any word beginning with the substring "trans"; *nin* matches any string containing the substring "nin".

• AND, NOT and OR have their obvious meanings. E.g., not trans* matches anything not containing a word beginning with "trans".

Another example of a search phrase might be

*trans* and not transcription

This matches sequences containing the substring "trans" but not the word "transcription". The *and *is important here; if left out, the phrase is interpreted as *trans* ornot transcription – and that matches *all *sequences! The Help pages show several more examples of search phrases (available from the front page).

In the **Classification criteria **selection box, the search may be restricted such that only transcription factors classified by certain criteria are searched.

**Figure 1:**
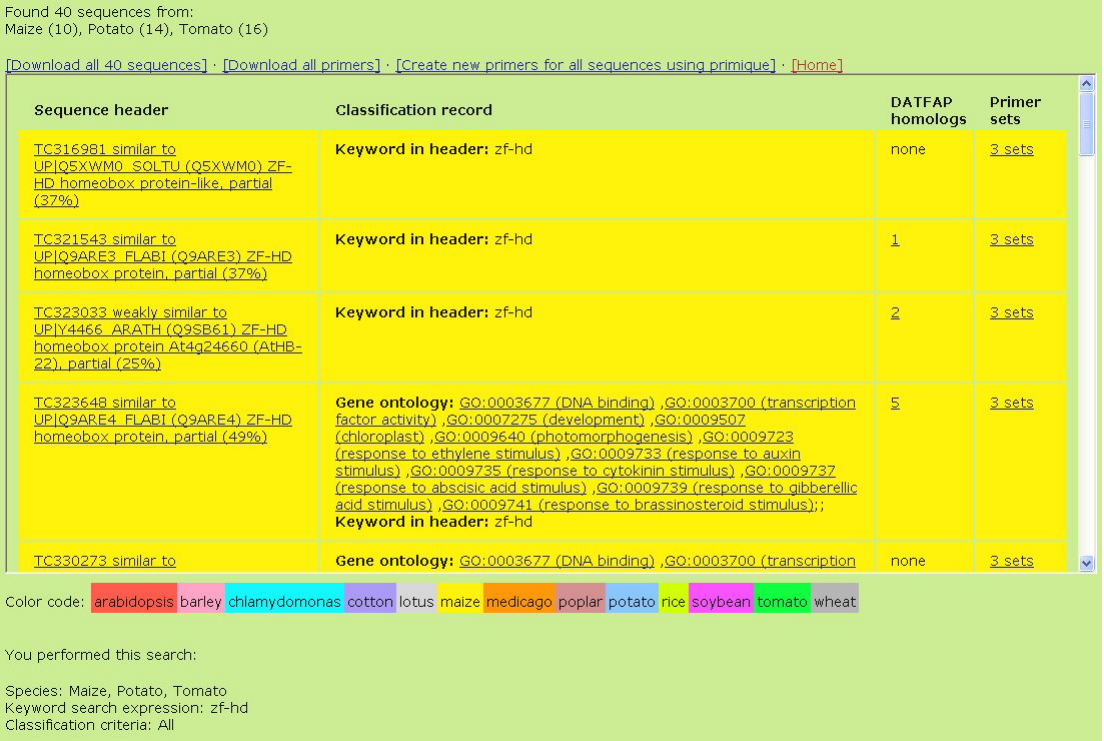
Showing the results of a search.

The sequences found in the search are presented in a scrollable table with four columns: **Sequence header**, **Classification record**, **DATFAP homologs **and **Primers**. Each species is represented by its own color, shown in a bar just below the table (Figure 1).

The classification record of a sequence states its reason for being in DATFAP; in other words, it states by which criteria this sequence has been classified as a transcription factor (see Materials and Methods).

In case primers are provided for a sequence, the **Primers **table column contains a link to a page giving details about them, including GC content, melting temperature, length and sequence of each primer, as well as the expected amplicon length and a score for the primer set (since primers are designed for ESTs and not full gene sequences, they may cross intron/exon boundaries. However, in practice this is probably not the reason why a primer pair might fail to yield a specific product, as observed in [13]). The score of a primer set depends on whether the individual primers have 1- or 2-mismatch alignments to any non-target sequences. Such "close match" alignments may be considered problematic since they represent potential mis-primings. A score of 3000 indicates that the primer pair has no 1- or 2-mismatch alignments to non-target sequences. Further details on the score can be found in [14].

**Figure 2:**
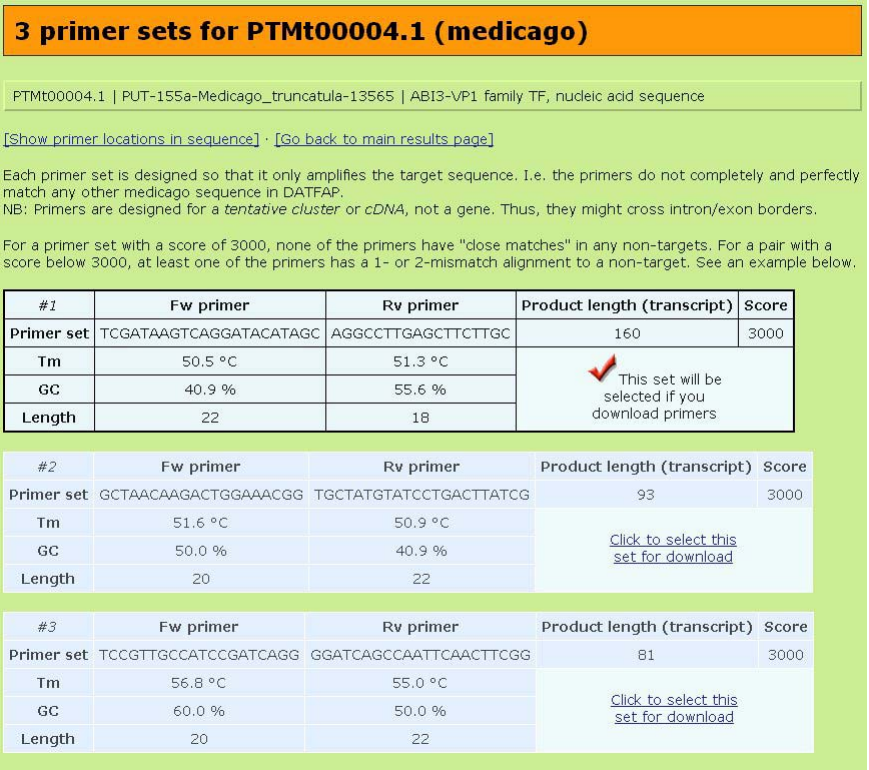
Three specific primer sets and their properties.

Up to three sets are suggested to allow the user an informed choice; any may be selected for download (Figure 2). The user can get a visual display of the locations inside the sequence of the selected primers by clicking a link.

All DATFAP primers are automatically designed using a standalone version of the tool primique [14] which designs primers for groups of sequences such that each primer pair uniquely and specifically amplifies its target sequence and no other sequence from the group. Thus, no primer pair in DATFAP has a perfect match among non-target DATFAP sequences from the target species.

The primer design has been carried out over three rounds for each species with different parameter settings optimized to deliver primers suited for Real-Time PCR (RT-PCR). The important ones of the first round are shown in Table 1.

**Table 1:** Selected parameter settings for round one in the primer design process.

Primer length	18 – 22 nucleotides
Product length	80 – 120 nucleotides
Primer melting temperature	58.0 – 60.0 °
Max. primer melting temperature diff.	2.0 °
Enforce G/C terminal	Yes
G/C content	40.0 – 60.0%
Max. base repeat	2 (e.g., GGG in a primer is disallowed)
Force specificity for both primers	Yes (i.e., none of the two primers has a perfect alignment to a non-target)

With these restrictive settings, the primers found will be good for high-throughput (multi-parallel) RT-PCR. In round two, the parameter settings are the same except a few which are relaxed according to Table 2.

**Table 2:** Parameter settings relaxed for round two in the primer design process.

Product length, round 2	80 – 160 nucleotides
Primer melting temperature, round 2	50.0 – 60.0 °
Max. base repeat, round 2	3 (e.g., GGG in a primer is allowed)

With this higher range of primer melting temperature, high-throughput RT-PCR is no longer the goal, but the primers will still be suited for single-transcript RT-PCR. If primers are not found for all sequences, the criteria are further relaxed in the final round three as summarized in Table 3.

**Table 3:** Parameter settings relaxed for round three in the primer design process.

Primer length, round 3	16 – 28 nucleotides
Product length, round 3	80 – 200 nucleotides
Max. primer melting temperature diff., round 3	4.0 °
Max. base repeat, round 3	4 (e.g., GGGG in a primer is allowed, although not in the 3'-end)

Thus, first all primers found in round 1 are stored. Then for round 2, primique is run with relaxed criteria on the sequences for which no valid primer sets were found in round 1. And finally, sequences still without primers are run through round 3. Some sequences are still without primers after round 3, most often because no primers can be found that align perfectly only to their target, or because the allowed product length is quite limited. The fraction of sequences for which primers could be found ranges from for 72.5% for *Arabidopsis *to 94.5% for poplar.

From the main results page (Figure 1), the user also has the opportunity to **Create new primers for all sequences using primique**. Clicking this link uploads all the result sequences to primique and lets the user decide all primer design parameter settings freely. The primers thus found are guaranteed to be specific among the uploaded sequences only, not among the entire set of same-species sequences from DATFAP as are the primers provided a priori with DATFAP.

The homologies between all sequences in DATFAP have been charted. This means that for any sequence resulting from a search, all its homologs can be displayed. For DATFAP purposes, two sequences A and B are defined to be homologous if and only if:

• blastn with *e*-value 10^-7 ^finds significant sequence similarity between A and B

• the total length of all regions in A that are similar to regions in B must be at least 60% of the total length of A, or vice versa.

Thus, in order for A and B to be defined as homologs, one must "cover" at least 60% of the other with high similarity. This measure naturally induces a graph structure over all sequences in DATFAP, called the *TF graph*, where the sequences are the nodes of the graph, and an edge between two nodes represents a homology relationship between them. Starting at any node in the graph, one can therefore follow its edges to reach its homologs – if it has any. DATFAP provides an easy means of navigating the graph this way, described below.

**Figure 3:**
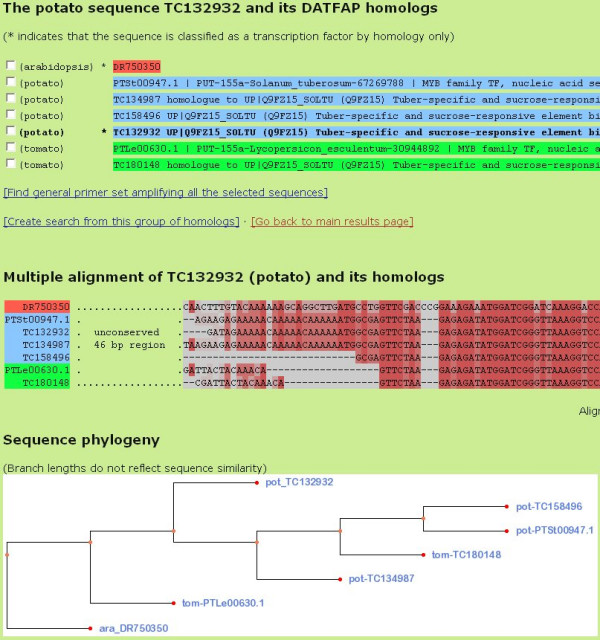
Multiple alignment and sequence phylogeny.

If a sequence has any DATFAP homologs, a link in the **DATFAP homologs **column (on the main results page, cf. Figure 1) takes the user to a page where a colored, multiple alignment of the sequence and all its homologs is displayed, as well as the sequence phylogeny deduced during the construction of the alignment using the UPGMA clustering method (Figure 3). All alignments are prepared by MultAlin [15] or, in the few cases that were too large for MultAlin, ClustalW [16]. The more red an alignment column is, the more sequences agree in this position (the details of this color scheme are given in the Help page on the DATFAP web site). Regions with low conservation are not shown in detail.

Phylogeny images are created by PhyFi [17]. By clicking the image, the user can open PhyFi in a new window, resize or otherwise manipulate the image and save it in a number of formats.

**Figure 4:**
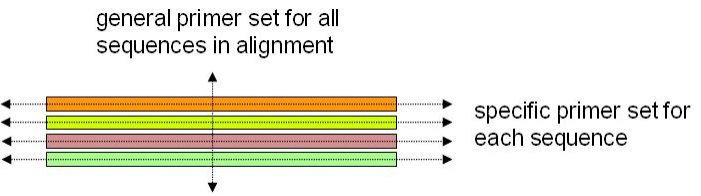
**An alignment of four homologs (colored bars).** Besides offering specific primers for individual sequences, DATFAP also allows the user to design a general primer set for any selected set of homologous sequences.

From the homology alignment page, there are two further links: 1) **Find general primer set amplifying all selected sequences**, and 2) **Create search from this group of homologs**. Next to each sequence header listed at the top of the page there is a small check box. Using these check boxes, the user may mark some of the sequences and design *one *general primer set targeting *any *of the marked sequences (using the tool PriFi [18]). If we think of the pre-designed, specific primer sets as being *horizontally *oriented (with respect to an alignment), this option allows the user to design primer sets which are *vertically *oriented (Figure 4).

**Figure 5:**
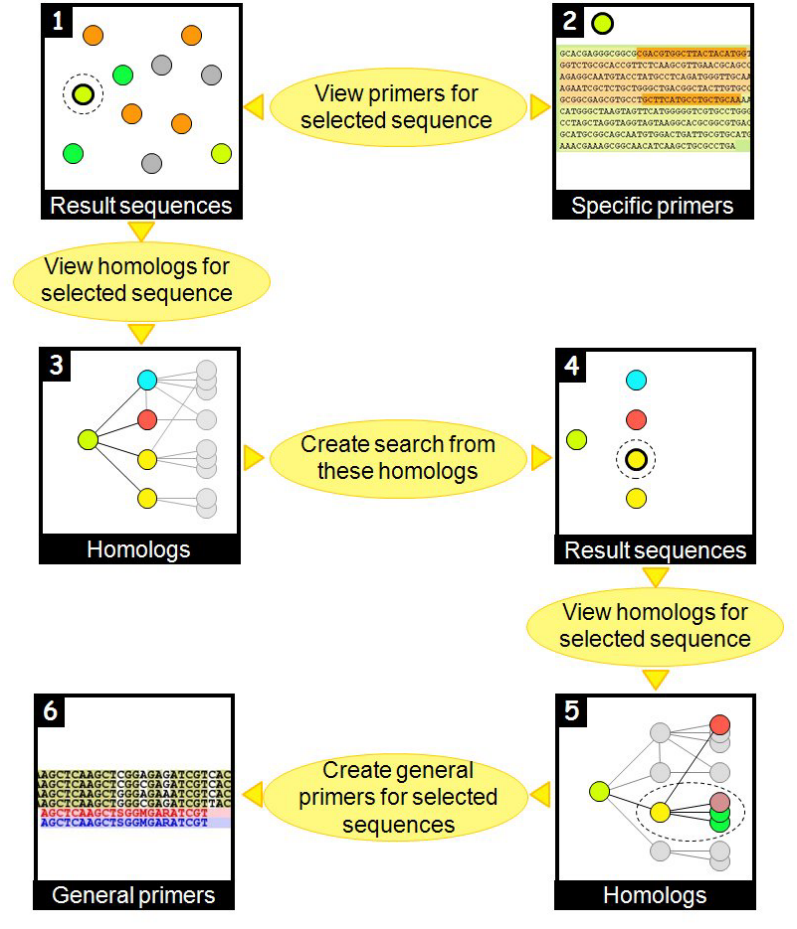
Using DATFAP.

The other link mimics performing a search from scratch which results in exactly the homologous sequences currently showing in the alignment. This corresponds to selecting the current "mother sequence" and its neighbors in the TF graph as the current sequences, and enables the user to view and download (FASTA format) this particular set of sequences, view and download (FASTA format) their specific primer sets, and view *their *homologs. This procedure is illustrated in Figure 5 which is explained next.

After the user has performed some search, frame 1 of Figure 5 shows the resulting sequences from *Medicago *(orange balls), cotton (purple balls), tomato (green balls) and rice (light green balls). The user may now choose to view the specific primers provided for, say, one of the rice sequences (frame 2). Next, going back to the main results page (frame 1), the user may click to view the homologs of the same rice sequence.

This leads to a page (frame 3) showing the rice sequence (light green ball) and its four DATFAP homologs from *Chlamydomonas *(ice blue ball), *Arabidopsis *(red ball) and maize (yellow balls). In fact, what is shown is a part of one of the connected components of the huge TF graph, namely the rice node and the four nodes it has edges to. Frame 3 shows other edges and nodes in the graph in shaded gray.

Now the user may choose to create a search from the group of five homologs currently showing. This leads to a new main results page (frame 4) with the rice, *Chlamydomonas*, *Arabidopsis *and maize sequences. Again, the user can choose to view the homologs of some selected sequence, this time a maize sequence (upper yellow ball), and frame 5 then shows this sequence and its five homologs from *Arabidopsis *(red ball), wheat (lilac ball), tomato (green balls) and rice (previously selected light green ball). Comparing to frame 3, this is simply a different part (subtree) of the TF graph with the yellow maize node as root.

Finally, the user can select some of the current sequences (rice, wheat and tomato sequences; yellow, lilac and green balls) and design a general set of primers targeting all of these sequences (frame 6). This is helpful if the user is studying homologs of the same transcription factor in several species: Then one such general primer set would suffice to amplify the gene in all species.

Since sequences are collected from several sources, duplicates do exist and have been removed from DATFAP. Two sequences are considered duplicates either if they are identical, or if they are at least 99.9% similar (according to blastn with *e*-value 10^-7^) while one is completely covered by the other (minus at most 10 nucleotides). Priorities are given to the classification criteria: if sequences A and B are considered duplicates while A originates from an external transcription factor database and B is solely in DATFAP because of homology to another DATFAP sequence, A is kept and B is removed since higher "transcription factor credibility" is given to external database origin than to homology.

## Conclusion

The primer pairs are designed according to stringent criteria regarding melting temperature, GC content etc., and first and foremost according to the principle that a primer pair must have a perfect alignment to its target sequence and to *no *other sequences from the same species in the database. Primer design and PCR is an experimental science, and no guarantees can be given that primers designed by a computer program will work, regardless of the program. Good algorithms show a very high success rate, though; e.g., in the extensive studies by Czechowski and colleagues [13] and Caldana and colleagues [19], 96% and 97% of all reactions yielded specific products, respectively. The primers of DATFAP are designed in a way which is similar to what was done in these studies. This author has designed several free, computerized, web-based primer design tools in the past according to exactly the same principles as those used for the DATFAP primers (e.g. [19,15]). One, PriFi, consistently has 25–50 users each day from more than 30 countries all over the world, most of whom are returning customers. In this author's view, the satisfaction of these users warrants the approach.

The projects described in [13,19] are Real-Time PCR platforms for *Arabidopsis *and Rice transcription factors, respectively, offering primers for the purposes of high-throughput expression profiling. Moreover, extensive experimental work utilizing the primers resulted in important insight and new knowledge regarding, e.g., organ-specific expression of certain genes. Such were not the primary goals of the DATFAP project. Through its suite of 13 species and easy means of searching and relating sequences within or between species, and through its predefined specific primers complemented by its option for creating cross-species primers, DATFAP is a general-purpose tool, hopefully paving the way for many different kinds of research centered around transcription factors.

DATFAP is a user-friendly, web-based, graphical resource freely available to anyone with an Internet browser. Equipped with a sophisticated search facility and specific primers for (almost) all sequences, DATFAP constitutes a valuable tool to researchers in all areas of plant molecular biology working with transcription factors. No other multi-species transcription factor database offers such easy inter- and intra-species navigation in the network of related transcription factors.

The DATFAP web site includes an elaborate Help page which illustrations, details and examples of the search facility.

## Availability and Requirements

Project name: "DATFAP: A Database of Primers and Homology Alignments for Transcription Factors from 13 Plant Species"

Project home page: 

Operating system(s): Platform independent (web-based)

Programming language: Python

Licence: Free to all non-commercial users

Any restrictions to use by non-academics: Licence needed.

## References

[B1] Miller G, Mittler R (2006). Could heat shock transcription factors function as hydrogen peroxide sensors in plants?. Ann Bot (Lond).

[B2] Toledo-Ortiz G, Huq E, Quail PH (2003). The Arabidopsis basic/helix-loop-helix transcription factor family. Plant Cell.

[B3] Memelink J, Kijne JW, van der Heijden R, Verpoorte R (2001). Genetic modification of plant secondary metabolite pathways using transcriptional regulators. Adv Biochem Eng Biotechnol.

[B4] Lee Y, Tsai J, Sunkara S, Karamycheva S, Pertea G, Sultana R, Antonescu V, Chan A, Cheung F, Quackenbush J (2005). The TIGR Gene Indices: clustering and assembling EST and known genes and integration with eukaryotic genomes. Nucleic Acids Research.

[B5] National Center for Biotechnology Information. http://www.ncbi.nlm.nih.gov/.

[B6] Guo A, He K, Liu D, Bai S, Gu X, Wei L, Luo J (2005). DATF: a Database of Arabidopsis Transcription Factors. Bioinformatics.

[B7] Riano-Pachon DM, Ruzicic S, Dreyer I, Mueller-Roeber B (2007). PlnTFDB: An integrative plant transcription factor database. BMC Bioinformatics.

[B8] Guo AY, Chen X, Gao G, Zhang H, Zhu QH, Liu XC, Zhong YF, Gu X, He K, Luo J (2008). PlantTFDB: a comprehensive plant transcription factor database. Nucl Acids Res.

[B9] Gene Ontology. http://geneontology.org/.

[B10] Altschul SF, Madden TL, Schaffer AA, Zhang J, Zhang Z, Miller W, Lipman DJ (1997). Gapped BLAST and PSI-BLAST: a new generation of protein database search programs. Nucl Acids Res.

[B11] Iida K, Seki M, Sakurai T, Satou M, Akiyama K, Toyoda T, Konagaya A, Shinozaki K (2005). RARTF: Database and Tools for Complete Sets of Arabidopsis Transcription Factors. DNA Research.

[B12] Riechmann JL, Heard J, Martin G, Reuber L, Jiang C, Keddie J, Adam L, Pineda O, Ratcliffe OJ, Samaha RR, Creelman R, Pilgrim M, Broun P, Zhang JZ, Ghandehari D, Sherman BK, Yu G (2000). Arabidopsis transcription factors: genome-wide comparative analysis among eukaryotes. Science.

[B13] Czechowski T, Bari RP, Stitt M, Scheible WR, Udvardi M (2004). Real-time RT-PCR profiling of over 1400 Arabidopsis transcription factors: unprecedented sensitivity reveals novel root- and shoot-specific genes. Plant J.

[B14] Fredslund J, Lange M (2007). Primique: automatic design of specific PCR primers for each sequence in a family. BMC Bioinformatics.

[B15] Corpet F (1988). Multiple sequence alignment with hierarchical clustering. Nucl Acids Res.

[B16] Thompson JD, Higgins DG, Gibson TJ (1994). CLUSTAL W: improving the sensitivity of progressive multiple sequence alignments through sequence weighting, position specific gap penalties and weight matrix choice. Nucl Acids Res.

[B17] Fredslund J (2006). PHYFI: fast and easy online creation and manipulation of phylogeny color figures. BMC Bioinformatics.

[B18] Fredslund J, Schauser L, Madsen LH, Sandal N, Stougaard J (2005). PriFi – Using a Multiple Alignment of Related Sequences to Find Primers for Amplification of Homologs. Nucl Acids Res.

[B19] Caldana C, Scheible W-R, Mueller-Roeber B, Ruzicic S (2007). A quantitative RT-PCR platform for high-throughput expression profiling of 2500 rice transcription factors. Plant Methods.

